# SNP microarray analyses reveal copy number alterations and progressive genome reorganization during tumor development in SVT/t driven mice breast cancer

**DOI:** 10.1186/1471-2407-12-380

**Published:** 2012-08-31

**Authors:** Christoph Standfuß, Heike Pospisil, Andreas Klein

**Affiliations:** 1, Bioinformatics, Technical University of Applied Sciences Wildau, 15745 Wildau, Bahnhofstraße, Germany; 2 Institute of Biochemistry, harité-Universitätsmedizin Berlin, 10117 Berlin, CCO, Charitéplatz 1, Germany

**Keywords:** Breast cancer, Genome reorganization, Copy number alteration, CNV, fragile sites, Cancer genomics, Tumorigenesis

## Abstract

**Background:**

Tumor development is known to be a stepwise process involving dynamic changes that affect cellular integrity and cellular behavior. This complex interaction between genomic organization and gene, as well as protein expression is not yet fully understood. Tumor characterization by gene expression analyses is not sufficient, since expression levels are only available as a snapshot of the cell status. So far, research has mainly focused on gene expression profiling or alterations in oncogenes, even though DNA microarray platforms would allow for high-throughput analyses of copy number alterations (CNAs).

**Methods:**

We analyzed DNA from mouse mammary gland epithelial cells using the Affymetrix Mouse Diversity Genotyping array (MOUSEDIVm520650) and calculated the CNAs. Segmental copy number alterations were computed based on the probeset CNAs using the circular binary segmentation algorithm. Motif search was performed in breakpoint regions (inter-segment regions) with the MEME suite to identify common motif sequences.

**Results:**

Here we present a four stage mouse model addressing copy number alterations in tumorigenesis. No considerable changes in CNA were identified for non-transgenic mice, but a stepwise increase in CNA was found during tumor development. The segmental copy number alteration revealed informative chromosomal fragmentation patterns. In inter-segment regions (hypothetical breakpoint sides) unique motifs were found.

**Conclusions:**

Our analyses suggest genome reorganization as a stepwise process that involves amplifications and deletions of chromosomal regions. We conclude from distinctive fragmentation patterns that conserved as well as individual breakpoints exist which promote tumorigenesis.

## Background

Cancer is known to be a disease involving dynamic changes affecting cellular integrity and cellular behavior [1]. To date, research has been focused on discovering gene expression profiles, alterations in oncogenes or tumor-suppressors, and genetic mutations; but since tumorigenesis is a complex multistep process, the transformation of a normal cell into a malignant tumor is not completely understood. It has been well known for decades that alternative pathways in cell transformation (e.g. changes in cell cycle, signal transduction, metabolism, immune response) via a stepwise progression to final malignant tumors exist [1-4].

In fact, genomic DNA is more stable than mRNA or proteins [5]. As a consequence of this, the focus on gene expression profiles may not completely reveal all genetic mechanisms of tumor development and progression. The alteration of chromosomal copy numbers is known to be a key genetic event in many well-studied diseases [5], such as Jacobsen syndrome [6], HIV acquisition and progression [7], systematic autoimmune diseases [8,9] and cancer phenotypes [10]. In normal human organisms more than 3% of the genome is known to be affected by copy number alterations (CNAs, also known as copy number variations - CNV) [11,12], whereas in mice the estimates differ from 3% [13] to 10.7% [14]. Significant efforts have been made to study CNAs in various organisms. Single nucleotide polymorphism (SNP) oligonucleotide microarrays and array comparative genomic hybridization (aCGH) allow for high-throughput analyses of CNAs. This enables the study of complex genomes and genetic events at a high resolution. Several studies have addressed CNAs in individuals from different mouse strains: Henrichsen et al. [14] and Cahan et al. [13] studied the impact of CNAs on the transcriptome, Cutler et al. [15] analyzed the gene content of inbred mouse strains, Graubert et al. [16] studied segmental DNA copy number alterations. Agam et al. [17] compared the CNAs found in the four mentioned studies with their own data and found significant differences. They show that 1.3% to 88.7% of the detected deletions and 2.1% to 100% of the gains are replicated from one study to the following ones. They infer that the reproducibility of these experiments depend on the array platform, the CNA detection algorithm and the protocols for platform design and hybridization. Moreover, microarray experiments in humans have revealed a connection between high amplified genes and gene expressions [18], and CNAs affecting well-characterized regions harboring tumor-suppressor genes in breast cancer and lung carcinoma [19]. Therefore, the development of highly reliable and high-resolution genetic analysis approaches as presented by Hannemann et al. [10], is of high therapeutical relevance. To investigate the impact of CNAs on gene expression, several studies used network-based approaches [20-23]. For example, the study of Jörnsten et al. [20] used a global model of CNA-driven transcription to model mRNA expressions with the help of CNAs.

**Figure 1:**
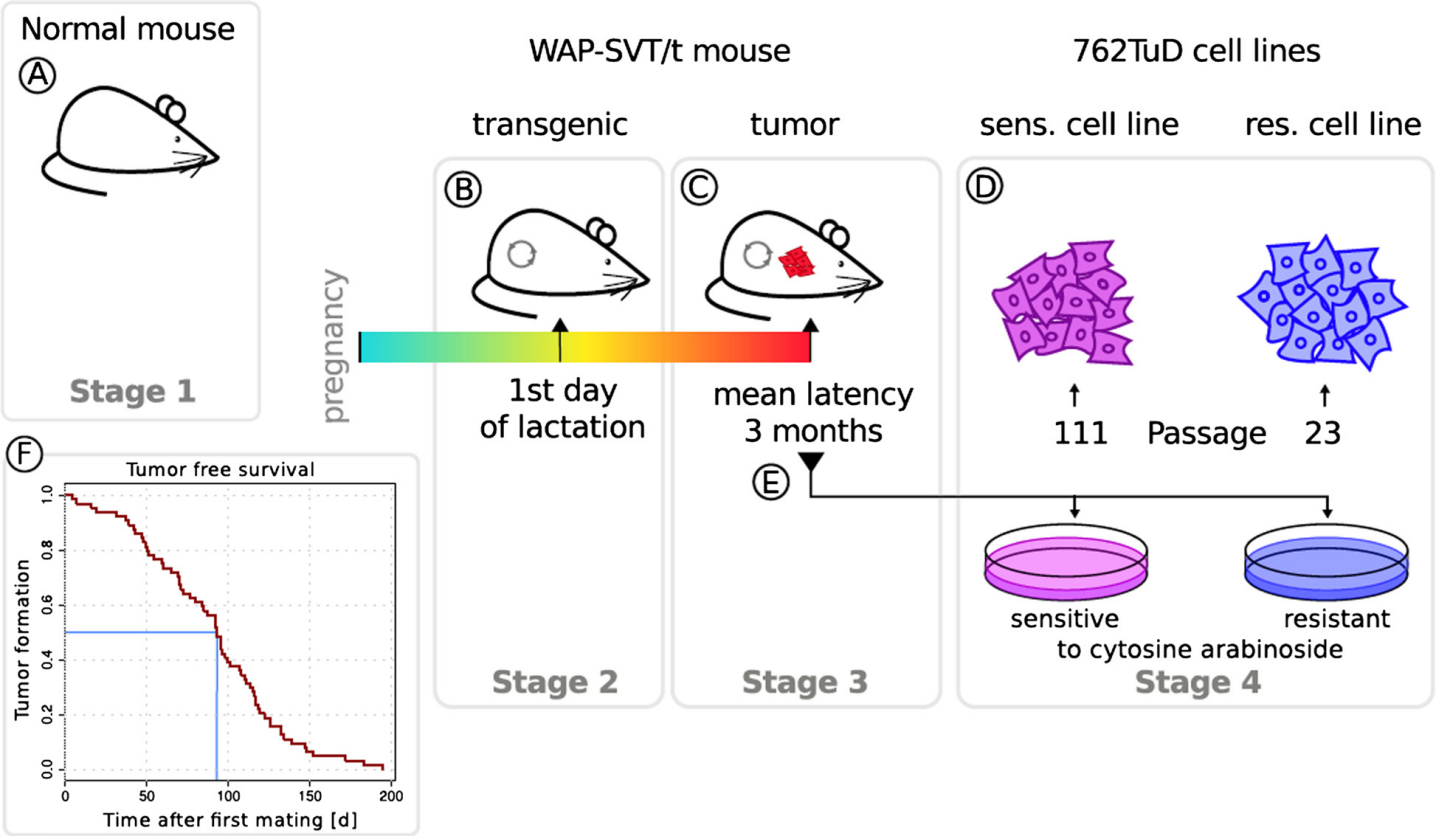
**Overview of mouse sample origin.** Mammary gland tissue samples from six NMRI mice were analyzed. Two normal samples were derived from two NMRI mice **(A)** and four mammary gland samples were derived from transgenic WAP-SVT/t mice. The transgenic samples **(B)** originate from these WAP-SVT/t mice, taken at first day of lactation. After the first lactation period all WAP-SVT/t transgenic mice had developed breast cancer. The two tumor samples were taken from these mice **(C)**. Additionally, two samples from two cell lines were used **(D)**. As described by Klein et al. [24] these two cell lines were established from mammary gland tumors **(E)**. The Kaplan-Meier survival curve for tumor-free survival after first mating is depicted **(F)** and the mean latency is marked in blue. A full version of the Kaplan-Meier curve can be found in Figure S1 (Additional file 2). The mouse picture was provided by Seans Potato Business and downloaded from Wikimedia Commons.

In the current study, we investigated the CNAs in a four stage tumorigenesis model. This model included copy number analyses in non-transgenic NMRI mice (normal; stage 1 in Figure 1) and in transgenic SVT/t mice: non-malignant hyperplastic mammary glands and breast cancers, as well as breast cancer derived cell lines (stages 2-4 in Figure 1, respectively). The WAP-SVT/t hybrid gene construct consists of the *Wap* (Whey acidic protein) promoter fused to the SV40 early coding region [3]. The WAP-SVT/t expression is selectively activated in breast tissue during pregnancy and continues after weaning. All female mice developed breast cancer after the first lactation period. We have established the 762TuD breast cancer cell line (termed sens. cell line) from a WAP SVT/t tumor, which has switched off SVT/t expression during the cultivation process and developed a p53 hotspot mutation (G242). The 762TuD cells are immortalized, malignant transformed and highly aneuploid. Additionally, we established a drug resistant 762TuD cancer cell line (termed res. cell line). The karyograms (via mFISH) of these two cell lines (named SVTneg1) are published ( [24], page 91). We focused our research on copy number analyses to compare the genomic alterations that occur during tumorigenesis. We addressed the question, whether common predisposed chromosomal breakpoints could be seen to promote malignant transformation. We can report a characteristic increase of copy number alterations from stage one to four (see Figure 1) in our model. Furthermore, we have identified continuous regions of copy number alteration (chromosomal segments) and found characteristic fragmentations. CNAs were compared on both the SNP probeset level and the level of continuous CNA regions (segments). Motif search was performed in hypothetical DNA breakpoint regions to find common motifs that may be coincident with a DNA break. The results of our model were compared to a model of PIK3CA-driven mammary tumors presented by Liu et al. [25].

## Results and discussion

To study the chromosomal aberrations and differences in gene expression at different stages of tumorigenesis, a mouse breast cancer model was applied (see Figure 1). To probe for chromosomal copy number alterations (CNAs) in this model we analyzed SNP arrays from mouse mammary gland epithelial cells. Eight samples were taken from two non-transgenic NMRI mice (normal) on the first day of lactation, two transgenic WAP-SVT/t mice on the first day of lactation, two WAP-SVT/t mouse breast cancer samples, and two WAP-SVT/t breast cancer derived cell lines (see Figure 1 and Table S1 in Additional file 1 for sample description). Copy number alterations were calculated from signal intensities detected by high-throughput single nucleotide polymorphism (SNP) microarrays.

**Figure 2:**
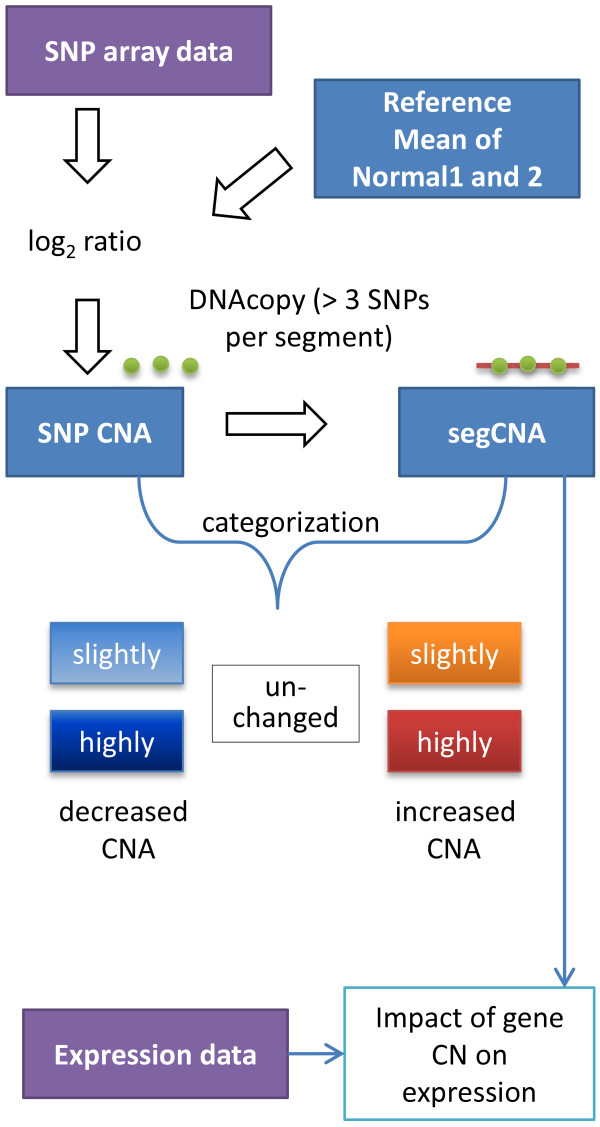
**Road map of the experimental approach.** We calculated the CNs from eight experiments (shown as purple box), built up a reference (mean signal intensity of Normal1 and Normal2) and determined the SNP CNAs for each sample against the reference. To assess the chromosomal segments we used the circular binary segmentation algorithm [26] with the restriction that adjacent SNPs with similar log_2_-ratios are necessary to form a segment (SNP CNAs are shown as green circles and the calculated segment segCNA is given as a red line). The SNP CNAs and segCNA values are categorized into five groups that are colored in the same manner as in Figure 3. Further, the SNP data were compared with gene expression data (given as a purple box) from the same samples.

For diploid organisms the usual copy number is expected to be two, and variations indicate chromosomal breakpoint events that are proposed to lead to phenotypic changes, e.g. to pathological aberrations. We searched in breakpoint regions for common sequence motifs. Additionally, we considered the gene expression in the context of chromosomal aberration. A road map of the experimental approach is given in Figure 2.

**Figure 3:**
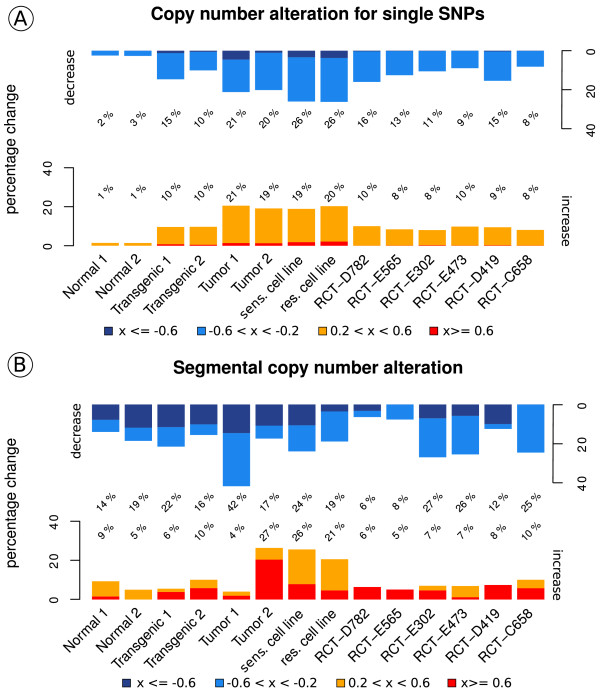
**SNP and segmental copy number alteration.** The percentage of SNP copy number values **(A)** and segmental copy number variations **(B)** was assigned to four groups and is illustrated. Log_2_-ratio values smaller than -0.6 are colored in dark blue, values ranging from -0.6 to -0.2 in light blue, log_2_-ratio values between 0.2 and 0.6 in orange and values greater than 0.6 in red. **(A)** Comparing the bars, one can see an increase in CNA from normal (∼ 4%) to transgenic (∼ 20 - 25%) and to tumor (∼ 40%). The copy number alterations in both SV40T/t cell lines are even higher compared to those in tumor (see Table S3A in Additional file 5 for entire CNA data). **(B)** In both normal samples about 76% of the calculated segments show no significant copy number alterations compared to the reference. An increase in CNA of 2% to 3% can be observed when comparing the transgenic samples to the normal samples, and by about 20% when comparing the tumor samples to the normal samples. The highest percentages of segCN were found in the Tumor1 and in the tumor sens. cell line. As observed in the number of segments the recurrent tumor samples form two groups with different magnitude of CNA (see Table S3B in Additional file 5 for entire segCNA data). A characteristic increase in segmental CN can be shown when comparing the stages of our model (see Figure 1).

Furthermore, the results presented in this work were compared to the data of six recurrent tumor samples published by Liu and coworkers [25].

### SNP copy number alteration

We analyzed 584,729 SNPs for each of the eight samples with the Affymetrix Mouse Diversity Genotyping array (see Yang et al. 2009 [27] for additional information), and calculated SNP copy number alterations (CNA) during tumorigenesis, which are indicators for chromosomal aberrations [10]. We added up the signal intensities for SNP alleles and compared the total intensities of all samples against a reference data set (mean signal intensity of both normal samples). For each SNP, CNA was computed by log_2_-ratios and all values in the range of −0.2<= × <=0.2 were considered as unchanged which corresponds to a fold change between 0.87 and 1.15. This indicates that not more than 30% of the cells carry the CNA. Compared to normal tissues a significant increase in the number of CNA was detected in the tumors (Welch two sample two-sided t-Test *p* = 8.43 ∗ 10^−8^). For visualization, log_2_-ratio copy number values of the SNPs were ranged into five groups to compare changes in different samples (see Figure 3A). We categorized the CNAs to unchanged (-0.2 <= × <= 0.2), slightly increased (0.2 < × < 0.6, orange), slightly decreased (-0.6 < × < -0.2, light blue), highly increased (× >= 0.6, red) and highly decreased (-0.6 < =× , dark blue). 96% of the SNP signal intensities were found to be unchanged in both normal samples (Normal1 and Normal2) (depicted in Figure 3A). These findings are in concordance with previously published studies [13,14]. In comparison, 10% of all SNP probeset intensities in the Transgenic2 sample show an increase in copy numbers (CNs), and 10% are decreased compared to the normal samples; in Transgenic1 the number of SNPs with a decrease in CNA is even higher (up to 15%). A further increase in CNA could be observed in both tumor samples. Here, approximately 21% of all SNPs show a decrease and an additional 21% show an increase in CN. The percentages in CN changes indicate a progressive increase of CNA from normal to transgenic and then tumor within our model. The highest percentage of CNA could be found in both cell line samples with a total change of 46.5% (sensitive cell line) and 45% (resistant cell line) of all SNP copy number values. Interestingly, comparable cell lines equally exhibit the most differentially expressed genes [3]. This reveals that considerable aberrations take place during cell cultivation.

For comparison we analyzed recently published data from Liu and colleagues [25], who have established a *PIK3CA*-driven breast cancer model conditionally expressing *PIK3CA*. CN analyses were carried out for six recurrent tumor samples with the Affymetrix Mouse Diversity Genotyping Array. A total change in CNA of about 26% can be identified in tumors RCT-D782 and RCT-D419; 16% to 21% of all SNPs in the remaining tumors show a copy number alteration. This is comparable to the changes detected in our transgenic samples. In fact, less changes in SNP copy numbers were found in the recurrent tumor samples than in both WAP-SVT/t tumor samples in our study. This may be explained by the differences in tumor development which became obvious in the mean latency of the tumor survival data: seven month for the *PIK3CA*-tumors in contrast to only three months in the WAP-SVT/t mice (see Kaplan-Meier survival curve in Figure 1F and supplemental Figure S1, Additional file 2).

### Detection of continuous CNA regions

The individual CNA of a single SNP may not be relevant or error-prone, hence we focused our research on genome reorganization. We addressed the purpose of continuous CNA detection on chromosomal regions and named these regions “chromosomal segments” (segCNA). The chromosomal segmentation of adjacent SNPs with similar log_2_-ratio values was calculated using the circular binary segmentation algorithm (CBS algorithm) introduced by Olshen et al. [26]. In both normal samples a similar number of about 70 distinct segments was detected. The number of calculated segments for the transgenic samples differed from 760 (Transgenic1) to 292 (Transgenic2) segments (see Table S2 in Additional file 3). A comparable difference in the number of segments was found in both cell line samples with 705 (sensitive cell line) and 354 (resistant cell line) segments calculated. In the tumors the number of segments in both samples also differ remarkable, by a factor of 7. 1,241 delimited segments were calculated in the Tumor1 sample whereas only 184 segments were found in the Tumor2 sample. This indicates an individual development of DNA reorganization for each sample during tumorigenesis. Although the SNP copy number alterations between both tumor samples were comparable, significant changes in chromosomal segmentation were found (see Tumor1 and Tumor2 in Table S2, Additional file 3). This can be explained by the CBS algorithm [26]. Only adjacent SNPs with a concordant signal intensity occur in contiguous regions of the chromosome. In contrast, the number of segments found in all six recurrent tumor samples differ from 31 segments in RCT-D782 to 85 segments in RCT-E472. Corresponding to our tumor samples we found two groups with significant differences in number of segments: group 1 having 31 to 42 segments in each sample, and group 2 having 68 to 85 segments per sample. This underlines the differences in both models and the individual development found for the copy number alteration analysis of individual SNPs. Two of the recurrent tumors (RCT-D782 and RCT-E565) of group 1 were found to retain a high abundance of active p-AKT and phospho-S6 ribosomal protein (p-S6RP); whereas two tumors (RCT-E472 and RCT-C658) of group 2 show a low abundance [25]. Although differences in segmentation were detected in both WAP-SVT/t tumor samples, about 9% of the calculated breakpoints in Tumor2 were also found in Tumor1 (see most inner circular track in Figure S4, Additional file 4). This indicates that even though the segmentation pattern may be different for each sample, they may share a common set of chromosomal breakpoints inducing similar reorganization patterns.

### Percentage of segment CN

As shown in Figure 3B the percentage of changed segment copy number (segCN) values in the tumor samples is remarkably higher than in the normal and the transgenic samples (by more than 50%). Interestingly, the amount of segments with a decrease in segCN is higher (value < -0.2) than those with an increase. This implies that deletion events are more frequent than amplifications (see Figure 3B and Table S3B in Additional file 5). The apparent increase in segCN of about 26% in Tumor2 is due to the small total number of 176 segments, compared to 1241 segments in Tumor1. The percentage of segmental copy number alteration of all recurrent tumor samples (published by Liu et al. [25]) is smaller than in the WAP-SVT/t tumor samples mentioned previously. Again, two groups can be identified. A variation in segCN was found for 13% to 20% of all segments in one group (RCT-D782, RCT-D565, RCT-D419), and in 33% to 35% of all segments in another. Moreover, as indicated by the different numbers of amplification and deletion events (see Figure 3B), it is obvious that tumor samples are heterogeneous.

### Segmentation patterns

**Figure 4:**
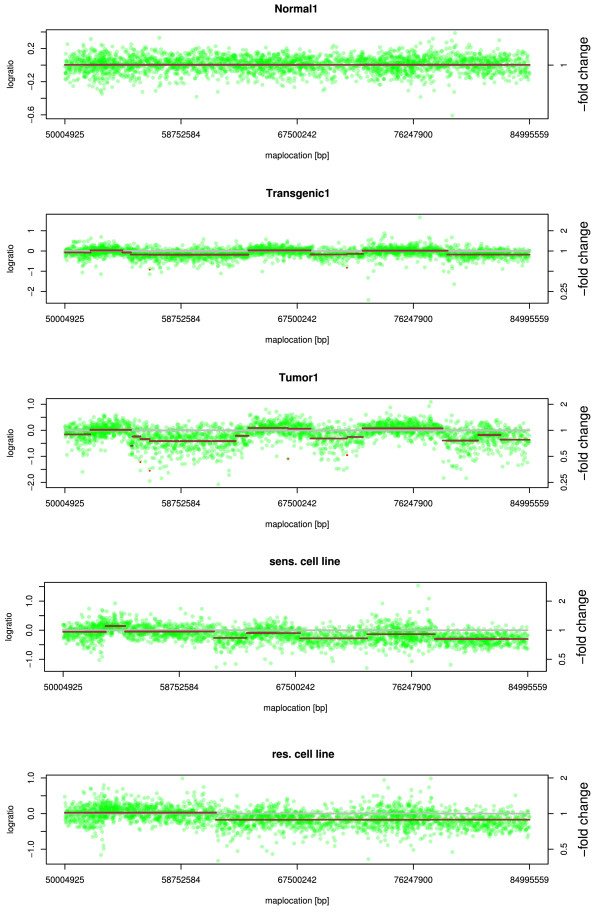
**Segmentation differences in developmental stages.** Fragmentation patterns which have frequently been observed are shown here; a section of chromosome 5 (55Mb to 85Mb) is taken as an example. Comparing Transgenic1 and Tumor1, one can find not only an increase in copy number alteration, but also a progressive fragmentation of previously found segments. These fragmentation patterns can be found in all WAP-SVT/t derived samples. The results for Normal2, Transgenic2 and Tumor2 were comparable (data not shown).

Log_2_-ratio SNP intensities were used to calculate the continuous regions of CNAs (called chromosomal segmentation), using the circular binary segmentation algorithm [26]. Characteristic patterns in segment copy number alterations (segCNAs) emerging in transgenic samples and further fragmented in tumor were found when analyzing the segmentation results. As illustrated in Figure S3 (see Additional file 6), a different segmentation of chromosome 6 within each sample was found. Additionally, an increase in segCNA can be found for each stage of the model. Not only differences in segment copy numbers themselves, but also different segmental positions (breakpoint positions) were detected when comparing the stages and samples. When taking a closer look at the Normal1, Transgenic1, Tumor1 and cell line samples, characteristic segmentation patterns can be observed. In Figure 4 a section of chromosome 5 (55 Mb to 85 Mb) is shown for each sample of all four model stages. No segmentation or breakpoints were found in the Normal1 sample; in contrast 14 segments with a log_2_-ratio value between -2 and 0.24 were detected in both transgenic samples. It is not only the case, that the number of segments is higher as summarized in Table S2, Additional file 3, or that new segments can be detected from the normal to the transgenic and the tumor samples, but also, the segments detected in tumor samples are mostly fragments from segments found in the transgenic sample (as illustrated in Figure 4). These segmentation patterns indicate predisposed chromosomal breakpoints. We think these breakpoints can be relevant as a prognostic parameter for tumor progression.

#### Comparison of CN studies

In comparing different CNA studies, one find only a weak overlap of segmental positions, segment length and copy number values [17]. Agam et al. [17] found 1,477 loss events and 499 gain events across seven mouse strains. 21 candidate regions of high-level DNA amplification were found in different carcinoma samples by Zhao et al. in 2004 [19]. Egan et al. [28] analyzed different mouse strains by tiling array CGH experiments and identified 38 CNAs for multiple probes and 23 segmental CNAs. Not only different segmentation algorithms and differences in probe hybridization, but also different types of microarray designs (aCGH, oligonucleotide) and different platforms may cause the problems. In their study Agam et al. [17] referred to the overlap of two sets of CNA between technical replicates. This overlap was compared to the overlap of CNAs called in animals of the same strain. Using the same algorithm and platform, they could show that more consistent results were produced by technical replicates rather than by biological ones.

### Segmentation and gene expression

To survey a possible correlation of gene expression and copy number variation, the method of direct comparison was used to evaluate the correlation of copy number and gene expression. We compared the impact of the copy number variation for different genomic regions on the resulting gene expressions for the top 500 differentially expressed genes for both normal, the Transgenic1, and the Tumor1 samples (see Methods). As shown in Table 1, 399 of 5,350 SNPs (see Table 1, underlined) in coding regions show a direct correlation, that implies a concordance of 7.5%: 358 SNPs within 330 up-regulated genes show an increase in copy number, and 41 SNPs show a decrease in copy number for 170 down-regulated genes. Altogether, few direct correlations between SNP copy number and gene expression were found. Analyzing the correlation between segmental copy numbers and gene expression (see Table 2), even a smaller concordance of 2.5% was found for amplified segments within up-regulated genes, and no concordance was found for deletions. Analyzing the association of CNA and gene expression in 44 primary tumors of 10 breast cancer patients, Pollack and co-workers [18] found that 62% of the highly amplified genes show moderate or high gene expression. Comparing the impact of CNAs to gene expression Lee et al. [29] summarize that it is no simple relation. They state that positive correlations can often be found (but not always), and other findings could be explained by other mechanisms, such as e.g. distant interactions and indirect regulation.

**Table 1:** Correlation of Gene expression and SNP copy number

**Gene expression**	**Number of genes**	**Number of SNPs with**			
		**Amplification**	**Deletion**	**No variation**	**Total**
up	330	358	104	3032	3494
down	170	121	41	1694	1856
		479	145	4726	5350

The correlation between the top 500 differentially expressed genes and the copy number of SNPs found within the coding regions was examined. For each up- or down-regulated gene the number of SNPs with an increase (amplification), a decrease (deletion) or no variation in copy number were counted. All in all, 5,350 SNPs were found within differentially expressed 500 genes; 7.5% of them had significant variations in SNP copy numbers (underlined). For merely 358 SNPs within the 330 up-regulated genes, an increased copy number was detected. For the 170 down-regulated genes only 41 SNPs were found to have a decrease in copy number.

**Table 2:** Correlation of Gene expression and segment copy number

**Gene expression**	**Number of genes**	**Number of segments with**			
		**Amplification**	**Deletion**	**No variation**	**Total**
up	330	8	0	305	313
down	170	2	0	213	215
		10	0	518	528

The correlation between the top 500 differentially expressed genes and the segment copy numbers found within the coding regions was examined. For each up- or down-regulated gene the number of segments with an increase (amplification), a decrease (deletion) or no variation in copy number values were counted. Interestingly, a decrease in segmental copy number was neither found for up-regulated nor for down-regulated genes. Only 8 of 313 segments associated to 330 up-regulated genes show an increase in segmental copy number, whereas the remaining 98.48% of the segments show no significant change.

**Figure 5:**
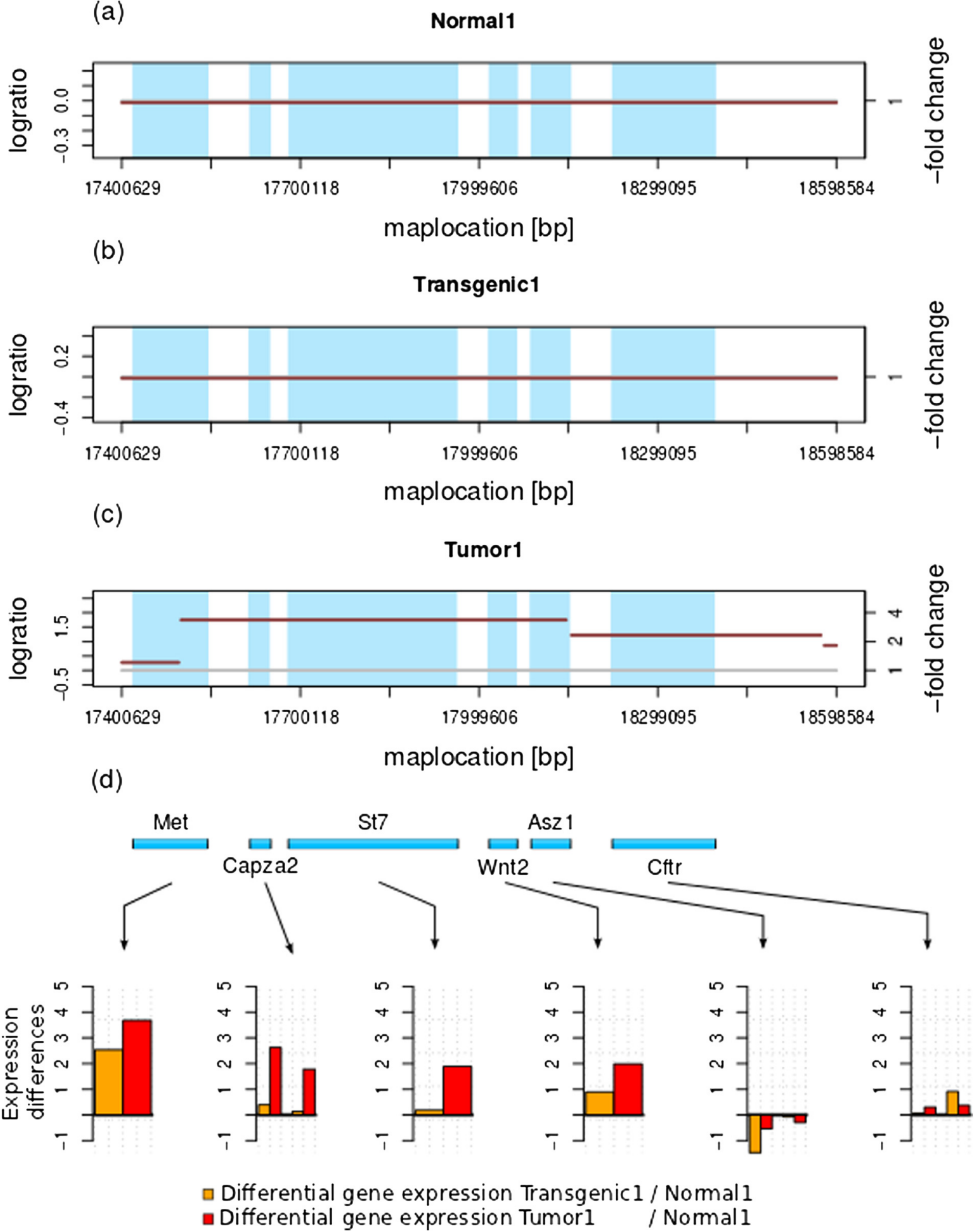
**Impact of copy number alteration on gene expression for a region of chromosome 6.** The impact of the copy number variation on the gene expression for a section of chromosome 6 (17.4 Mb to 18.6 Mb) is shown here. Six genes showing a significant up-regulation in gene expression are located within this region, including *Met*, *Capza2*, *St7*. The segmentation in the normal, the transgenic and the tumor samples are shown in subplots **a**, **b** and **c**, respectively. The gene positions are illustrated in **(d)** and in each plot, illustrating the copy number variation. One breakpoint was found within the *Met* gene resulting in a segment with more than a 3.5-fold amplification in CN.

However, a few examples of direct correlation to gene expression can be identified in some chromosomal regions. As an example, a region of chromosome 6 in Normal1 (a), Transgenic1 (b) and Tumor1 (c) is depicted in Figure 5, showing the chromosomal region from 17.4 Mb to 18.6 Mb. Four segments with a high copy number alteration in tumor (c) and 6 protein coding genes (d) affected by CNA were found within this region. Comparing the gene positions to the calculated breakpoints, the first chromosomal breakpoint could be identified within the *Met* gene, the second between the *Asz1* and the *Cftr* gene and the third around 18.46 Mb. Not only was an increase in copy number variations for three segments detected, but also a significant up-regulation for *Met* (about 3.8), *Capza2* (1.8 to 2.7) and *St7* (about 1.9) was detected. *Met* is a well known proto-oncogene which shows a high expression in different tumor entities [30], e.g. in breast cancer [31,32]. Even though, an increased segCN was computed for *Capza2*, *St7*, *Wnt2* and *Asz1*, a significant up-regulation in gene expression was found for *Met*, *Capza2* and *St7*. Neither the CNA within this region nor the differential gene expression of the listed genes can be found any of the other samples. Modeling transcriptional effects of CN in glioblastoma, Jörnsten et al. [20] state that some CNA-mRNA associations may be erroneous since CNAs often span multiple genes. Using CNA-driven networks they found 512 associations between gene expression and CNA in the glioblastoma data of 186 patients. Applying copy number eQTL analysis (eQTL - mapping of quantitative trait loci regulating gene expression) to 20,145 mouse genes in their study, Ahn et al. [23] showed significant overlaps with existing networks and found that significant genes were highly connected as compared to non-essential genes. At the moment we are not able to apply network-based methods to our data due to the small number of experiments. We will however in future research address molecular networks of tumor progression in our model.

#### qPCR verification

We reviewed the previously described CN amplification by qPCR analyses for the unamplified region (chr6:17.3MB-17.5MB, log_2_-ratio = 0.3 in Tumor1) including parts of the Met gene and the amplified region (chr6:17.5MB-18.14MB, log_2_-ratio = 1.75). One primer pair was located within the unamplified segment, two pairs within the amplified segment. In Figure S2 (see Additional file 7) the results of qPCR analyses of Normal1, Normal2, Tumor1 and Tumor2 are shown. Only in Tumor1 an amplification was found, for both primer pairs up to six-fold within the region expected to be amplified. Compared to the normal samples and the Tumor2 sample, a slight amplification was detected within the region expected to be unamplified. This is reflected in the small log_2_-ratio change of 0.3 (1.23-fold) detected. Both results are in accordance with the calculated segment intensity values from our CNA data (see Figure 5).

### Motif search and repeats

**Figure 6:**
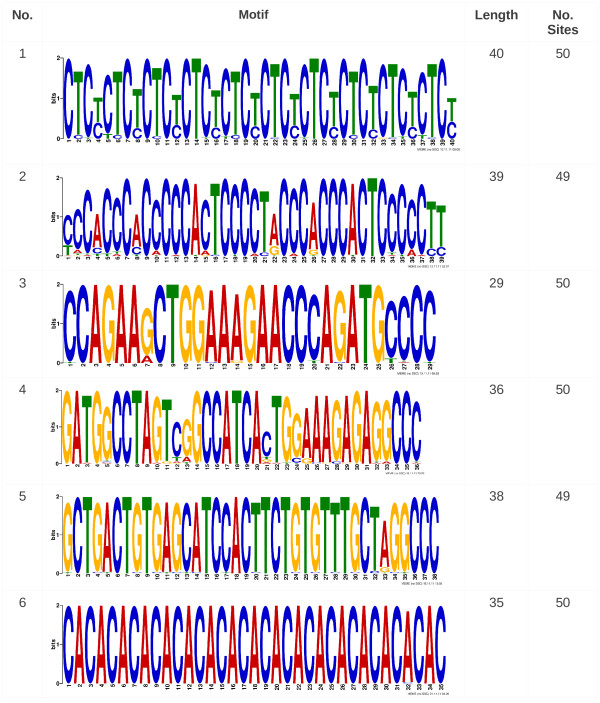
**Motifs.** The six motifs detected in 285 inter-segment regions of Tumor1 are presented. The lengths of the motifs vary from 29 to 40 bp with 49 and 50 common sites.

Segmental positions depend on the chromosomal location of the SNPs, but the distance between two adjacent segments may span about 4kb on average. These inter-segment regions (ISRs) comprise hypothetical breakpoints but the exact positions were not detectable. Hence, motif discovery was performed (with MEME Suite [33]) for motif identification in hypothetical breakpoint sequences. We present here six motifs detected within the 285 inter-segment regions of Tumor1 (see Figure S4 in Additional file 4 for motif positions). As shown in Figure 6, motif 1 consists of multiple CTC[T/C] repeats and can be found in at least 50 sites. As with motif 1, motif 6 consists of multiple [CA]^*n*^repeats with a total length of 39 bp. The motifs show further repeats besides the previously mentioned ones, eg. [C]^3^ and [C]^5^ in motif 2 or GG[C/A]^2^ in motif 4. These simple repeats have been confirmed by a previous study by Puttagunta et al. [34]. This study revealed that simple repeat sequences may be involved in chromosome breaks. Most of these simple repeats consist of a multiple sequence of dinucleotide repeats, like [CT]^*n*^[34] and [TA]^*n*^[35] repeats. Repeats of [TCTG]^*n*^and [GTCTCT]^*n*^[34] have also been observed within chromosomal breakpoints. Ruiz-Herrera et al. also showed the correspondence between fragile site location and the positions of evolutionary breakpoints [36]. As stated by Ruiz-Herrera, microsatellites are known to be an additional underlying mechanism behind chromosomal instability, characterizing some fragile sites in human DNA, and in constitutional human chromosomal disorders. Not only are microsatellites repeats of varying length, but they have also been found to be particularly AT-rich [37]. Furthermore, palindromic AT-rich repeats are found to be related to human chromosomal aberrations [38,39]. We determined the associated GO terms (Gene Ontology) of the six motifs using GOMO [40] (Gene Ontology for Motifs, from MEME suite [33]); the top GO term predictions are listed in Table 3. The association of the term “positive regulation of transcription from RNA polymerase II promoter” is very common to motifs 1 and 5. Motif 1 was also identified to be associated to a “negative regulation of transcription from RNA polymerase II promoter”. Interestingly, only a cellular component association was found for motif 3 and no association was found for motif 4. Additionally, three of six motifs were found to be associated to “transcription factor activity”. Comparing the motifs found within the inter-segment regions (ISRs), seventeen matches were computed searching the Uniprobe mouse database with TomTom [41]. Most motif matches were found for motif 2, including Zinc finger protein motifs, growth factor response motifs and homeodomains. In summary, an association to DNA, RNA and protein interaction as well as an influence on transcriptional regulation can be found for four of the six previously presented motifs. These motif characteristics are indicated not only by motif associations to GO terms but also by motif matches to validated and well known motifs. Motifs having neither a GO term prediction nor matching known motifs, may still by further analyses be shown to contribute to breakpoint prediction.

**Table 3:** Motif annotations

**Motif**	**GO term predictions**	**Motif match**
1	**BP** - positive regulation of transcription	*no match*
	from RNA polymerase II promoter	
	**BP** - transcription	
	**BP** - negative regulation of transcription	
	from RNA polymerase II promoter complex	
	**MF** - transcription activator activity	
2	**MF** - transcription factor activity	Zfp740, Zfp281,
	**MF** - sequence-specific DNA binding	Sox13, Sp4,
	**BP** - transcription	Pitx3, Smad3,
	**BP** - inner ear morphogenesis	Egr1, Ascl2,
	**BP** - proximal/distal pattern formation	Zfp410
3	*no prediction*	Spdef, Tcfe2a
4	*no prediction*	Zbtb3
5	**MF** - sequence-specific DNA binding	*no match*
	**MF** - transcription factor activity	
	**BP** - positive regulation of transcription from	
	RNA polymerase II promoter	
	**MF** - calcium ion binding	
6	**MF** - receptor binding	Gm397
	**BP** - axon guidance	
	**BP** - positive regulation of immune response	
	**BP** - defense response	

Motifs found by motif search in 285 segments of the Tumor1 sample are depicted in Figure 6. GO term associations using GOMO [40] and the motif matches against the UniProbe [42] database using TomTom [41] were computed for each motif. In cases of motif 1, motif 2, motif 5 and motif 6 Gene Ontology term associations were found. Biological processes are abbreviated by BP, molecular functions by MF. Nine motifs of the UniProbe database match motif 2 and only two UniProbe motifs match motif 3.

## Conclusions

In this work we study the CNAs of a four stage tumorigenesis model. Our model includes copy number analyses in a normal, in a transgenic, and in a tumor phenotype as well as in tumor-derived cell lines. We analyzed the copy number (CN) of mouse mammary gland epithelial cells and compared their gene expression to the copy number alterations detected. Here, we demonstrated a stepwise increase in fragmentation of mouse chromosomes during tumorigenesis with non-random fragmentation patterns within each stage of our model. Nearly 10% of all breakpoints detected in the Tumor2 sample were found to be common with the Tumor1 sample. This indicates that individual breakpoints as well as common breakpoint patterns contribute to tumor progression. Further analyses will have to confirm the impact of these common breakpoints on tumorigenesis. The distinctive fragmentation showing a stepwise increase of copy numbers suggest predisposed or conserved breakpoints which promote oncogenesis. The limitation of this work was the small number of samples for the comparison of copy numbers and gene expression, making it hard to determine the exact correlation between them, also making the determination of conserved or common breakpoints within one stage difficult. Therefore, further experiments on a larger number of samples will be undertaken to find a subset of breakpoints or chromosomal regions common within a stage. Animal models provide a reliable basis for further experiments. Samples from transgenic SVT/t mice during the first lactation period are comparable to early tumor stages in human breast cancer [43]. A goal of this work was to discriminate between early and late genomic changes in tumor development. The profound identification of early stages in breast cancer would be helpful for diagnosis and could influence the therapeutic decisions. Further, we might detect a chronology in genomic reorganization during tumorigenesis. Nevertheless, a large number of experiments is necessary if one is to study the impact of CNAs and breakpoints on gene expression differences during tumor development. The six motifs identified in inter-segment regions (ISRs) show a significant appearance in more than 40 different ISRs. Two of these six motifs were found to have no GO term associations, but they match known motifs from the UniProbe database. Two other motifs found within the ISRs match no known motifs of the UniProbe, but an association to biological processes and molecular functions could be predicted. Further analyses have to be made, analyzing the exact function of these motifs in ISRs and their effect on CN and chromosomal breakpoints.

## Methods

### Material

Mammary gland tissue samples from six NMRI mice were analyzed. Two samples originated from normal non-transgenic mice, and four from WAP-SVT/t transgenic mice (see Figure 1). The two transgenic samples were derived from WAP-SVT/t mice on the first day of lactation. Moreover, two breast cancer samples originated from WAP-SVT/t mice that had developed cancer after the first lactation period. Additionally, two samples were derived from the 762TuD cell lines as described in the work of Klein et al. [24]. The cytosine arabinoside sensitive sample SVTneg1 (CAs) was in passage 111 and the cytosine arabinoside resistant sample SVTneg1 (CAr) was in passage 23 when DNA was taken for analyses. The data have been deposited in GEO database [44] and are accessible through GEO Series accession number GSE35873 ( http://www.ncbi.nlm.nih.gov/geo/query/acc.cgi?acc=GSE35873). The induction of tumor formation by SV40-T-antigen synthesis was tested in a previous work by Santarelli et al. [45].

For further comparison, six recurrent tumor samples of PIK3CA-driven mammary tumors provided by Liu et al. [25] were used for the analyses (data available at NCBI GEO database [44], accession number GSE27691).

### Mouse SNP analyses

DNA was extracted using Purelink Genomic DNA Kit (K1820, Invitrogen) in accordance with the manufacturer’s protocol. The genotyping analyses were carried out at Atlas Biolabs GmbH (Berlin, Germany) using Affymetrix Mouse Diversity Array (MOUSEDIVm520650) [see supplement for protocol, Additional file 8. The array design was described by Yang et al. in 2009 [27]. Normalization and allele summarization were performed with the BRLMM-P algorithm provided by the Affymetrix Power Tools Software Package (version 1.14.3.1). To compare the total signal intensity distributions of all samples, intensities of both alleles for each SNP were added up. Copy number alteration (CNA) for each SNP was computed as log_2_-ratios of each sample and a reference dataset. The reference for each SNP was calculated as the mean signal intensity of both normal samples (Normal1 and Normal2). In the case of the six recurrent tumor samples the ratio was computed using the normal sample provided by Liu et. al. [25].

### Segmentation analyses and motif finding

All statistical analyses were performed using R (version 2.14). Differences in copy number (CN) and segmentation of each chromosome were calculated with the DNAcopy package (version 1.28.0) of Bioconductor (version 2.9) [46], using log_2_-ratio values. The DNAcopy package implements the circular binary segmentation algorithm introduced by Olshen et al. [26]. Continuous CNA regions (segments) were predicted finding a ’change-point’ between two groups of SNP intensity values according to their common distribution function. The parameters of the significance level *α* and the standard deviation *SD* were tested to assess the number of resulting segments (data not shown). Here the parameter settings of *α* = 0.001, *SD* = 0.5 and “sd.undo” were used. Motif search was performed in inter-segment regions (ISR) of the Tumor1 sample using the MEME Suite [33]. To enhance the significance, only inter-segment regions of two adjacent segments with a difference in segment mean of at least 0.8 were analyzed. The MEME parameters were set to a minimum motif width of 15 bp and a maximum width of 40 bp. Motifs found within the ISR were annotated using GOMO [40] and compared to known motifs of the UniProbe database [42] using TomTom [41].

### Gene expression analyses

RNA was extracted from frozen tissue segments with RNAzol (PeQLab, Biotechnology GmbH) in accordance with the manufacturer’s protocol. RNA was hybridized to Affymetrix’s Mouse Expression Set 430 A; chips were scanned with the GeneChip Scanner 3000 and VSN normalization was applied to the gene expression data for normalization. Gene expression data (published by Klein et al. [43]) can be found on NCBI Gene Expression Omnibus database [44] (GEO series accession number GSE6772; see Additional file 1 for sample accession numbers). Differentially expressed genes were determined based on the false discovery rate adjusted p-value (FDR p-value), using the limma package [47] (version 3.10.1) of Bioconductor. For comparison of the gene expression and the copy number variation, the 500 top-ranked differentially expressed genes between the two normal and the two tumor samples were computed. It was analyzed whether an increase or decrease of a gene CN influences the gene expression.

### Quantitative real-time polymerase chain reaction

DNA samples from two non-transgenic NMRI mice on the first day of lactation and two WAP-SVT/t tumor samples were used for quantitative PCR analysis. Quantitative real-time polymerase chain reaction (qPCR) was performed on optical grade PCR plate (BioRad Laboratories, Munich, Germany) using a BioRad iQ iCycler Detection System (BioRad Laboratories). All qPCRs were performed in triplicate in a total volume of 20 *μ*l, containing 15 ng of gDNA sample, 20 nmol of each primer, and 10 *μ*l of SensiFAST SYBR Lo-ROX Kit (Bioline, Luckenwalde, Germany). Baseline setting, Ct values and efficiency of PCR reactions were determined with the help of LinRegPCR version 12.16 [48,49]. Relative quantities of the gene to be studied were normalized to glyceraldehyde 3-phosphate dehydrogenase quantities. Each experiment was carried out in triplicate. The following primers were used for qPCR analysis: for the unamplified region Met_ua_s 5’-TGCTTGGTGACTTTGGTGTGGT-3’ and Met_ua1_as 5’-AGCAGGCAGAAATGCGTGAAAGT-3’; for the amplified region Met_am_1_s 5’-ACGTGGAGTTCAGCAGCAATCTGT-3’ and Met_am1_as 5’-TGGCTTGGGATTAGGGCTGTTCT-3’ as well as Met_am2_s 5’-CCTCCAGCACGGGATTCAACCA-3’ and Met_am2_as 5’-TGACTACATGCCGCGCCTAAC-3’.

### Survival analysis

We analyzed the time it took for tumors to develop in 64 female mice. Time was measured from first day of mating until the finding of a tumor; after a tumor was found the mice were euthanized. The Kaplan-Meier survival curve was computed using the R package Survival (version 2.36-10).

### Array annotations and genomic information

SNP array annotations of release 31 were downloaded from Affymetrix’s website and used for SNP copy number analyses and segmentation analyses. Mouse DNA sequences were downloaded from Ensembl [50] (release 65, Mouse Genome Assembly NCBI m37).

### Animal care

All animal experiments were carried out in accordance with the protocols of the animal care committee of the Senate of Berlin.

## Abbreviations

aCGH: Array comparative genomic hybridization; CA: Cytosine arabinoside; CN: Copy number; CNA: Copy number alterations; GO: Gene Ontology; ISR: Inter-segment region; PIK3CA: phosphoinositide-3-kinase; segCNA: Segmental copy number alteration; qPCR: Quantitative PCR; SNP: Single nucleotide polymorphism.

## Competing interests

The authors declare that they have no competing interests.

## Authors’ contributions

HP and AK initiated and designed the study. The statistical analyses were performed by CS and supervised by HP. The laboratory work was performed by AK. CS drafted and HP and AK edited the manuscript. All authors read and approved the final manuscript. HP and AK share the senior-authorship.

## Pre-publication history

The pre-publication history for this paper can be accessed here:

http://www.biomedcentral.com/1471-2407/12/380/prepub

## Supplementary Material

Additional file 1 **Sample description.** Table S1: The sample names used in this publication are listed. GEO accession numbers for each experiment can be found in this table. For the Transgenic2 (183T8) sample no gene expression data was available. Click here for file

Additional file 2 **Kaplan-Meier curve.** Figure S1: This figure illustrates the Kaplan-Meier curve. The x-axis depicts the duration from the first mating and the finding of tumor formation. All mice were euthanized as soon as a tumor was found. All 64 mice developed breast cancer within less than 200 days after their first day of pregnancy. In fact, about 60% of the animals showed a tumor formation within the first 100 days. Click here for file

Additional file 5 Log2-ratio distribution. Table S3: (A) and (B): Tables listing the alteration of single SNP log2-ratio (as shown in Figure 3A) and the alteration of segment log2-ratio values (as shown in Figure 3B). Click here for file

Additional file 3 Number of segments computed for each sample. Table S2: This table lists the number of segments calculated for each chromosome in each of the 14 samples. Click here for file

Additional file 4 Copy number alteration and motif position in Tumor1 sample. Figure S4: Copy number alterations are depicted in the outer cirular plot. The five inner circular plots illustrate the motif positions of motif 1 (blue), motif 2 (orange), motif 3 (green), motif 4 (red), motif 5 (purple) and motif 6 (grey). Thicker lines illustrate a short distance of two motif positions. Common breakpoints of Tumor2 and Tumor1 samples are illustrated in the most inner circular plot. Click here for file

Additional file 6 Segmentation in different samples. Figure S3: Different segmentation results for chromosome 6 in all samples is depicted. Comparing Normal1 to Transgenic1 and to Tumor1, one can see an increase in both the fragmentation and the copy number. Comparable alterations can also be found in both SV40 cell line samples. By comparison, the Transgenic2 and Tumor2 samples show less fragmentations. Interestingly, even more segments can be identified in the Transgenic2 sample than in Tumor2. Click here for file

Additional file 7 Plot of qPCR results. Figure S2: Barplot illustrating the qPCR results for the three previously mentioned regions of chromosome 6. Click here for file

Additional file 8 Genotyping Protocol. Protocol of genotyping analyses. Click here for file
