# Supplementary figures and images for: SNP microarray analyses reveal copy number alterations and progressive genome reorganization during tumor development in SVT/t driven mice breast cancer
Source: BMC Cancer. 2012 Aug 31;12:380. doi: 10.1186/1471-2407-12-380 (PMC3534550; doi:10.1186/1471-2407-12-380)

# Tumor free survival

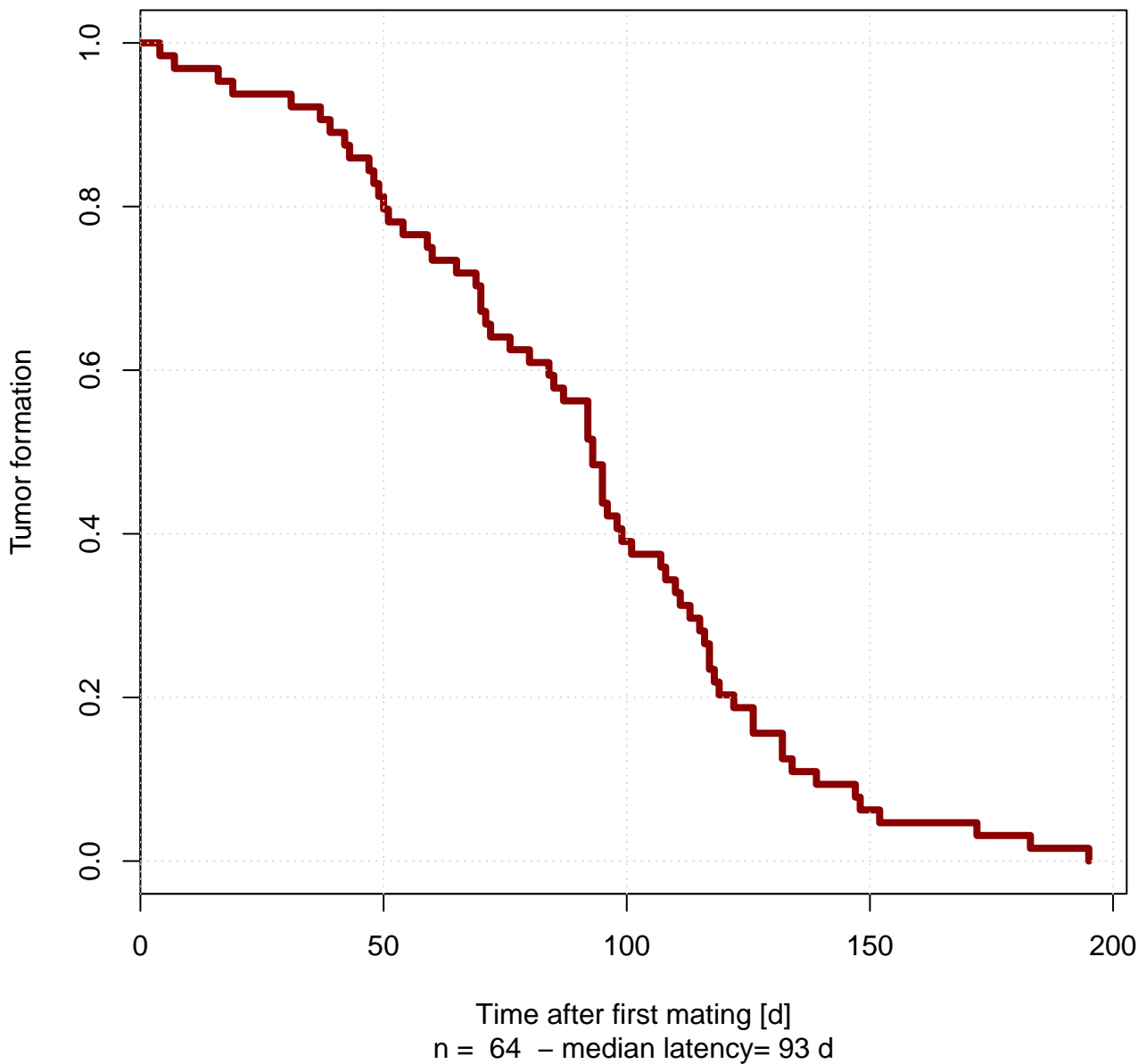

Supplement: Additional file 1 — Sample description. Table S1: The sample names used in this publication are listed. GEO accession numbers for each experiment can be found in this table. For the Transgenic2 (183T8) sample no gene expression data was available. [file 1471-2407-12-380-S1.pdf]

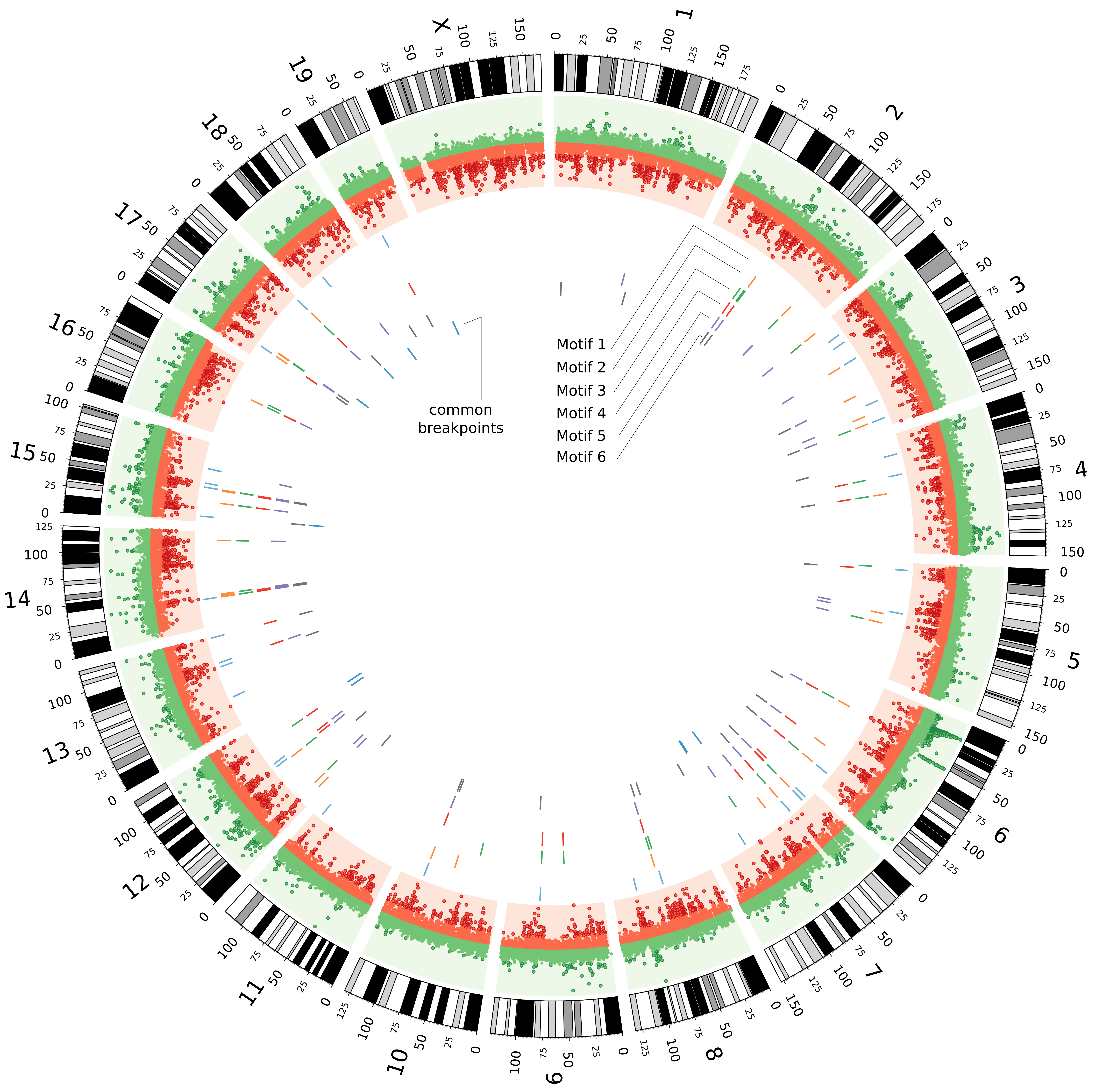

Supplement: Additional file 5 — Log2-ratio distribution. Table S3: (A) and (B): Tables listing the alteration of single SNP log2-ratio (as shown in Figure 3A) and the alteration of segment log2-ratio values (as shown in Figure 3B). [file 1471-2407-12-380-S5.pdf]

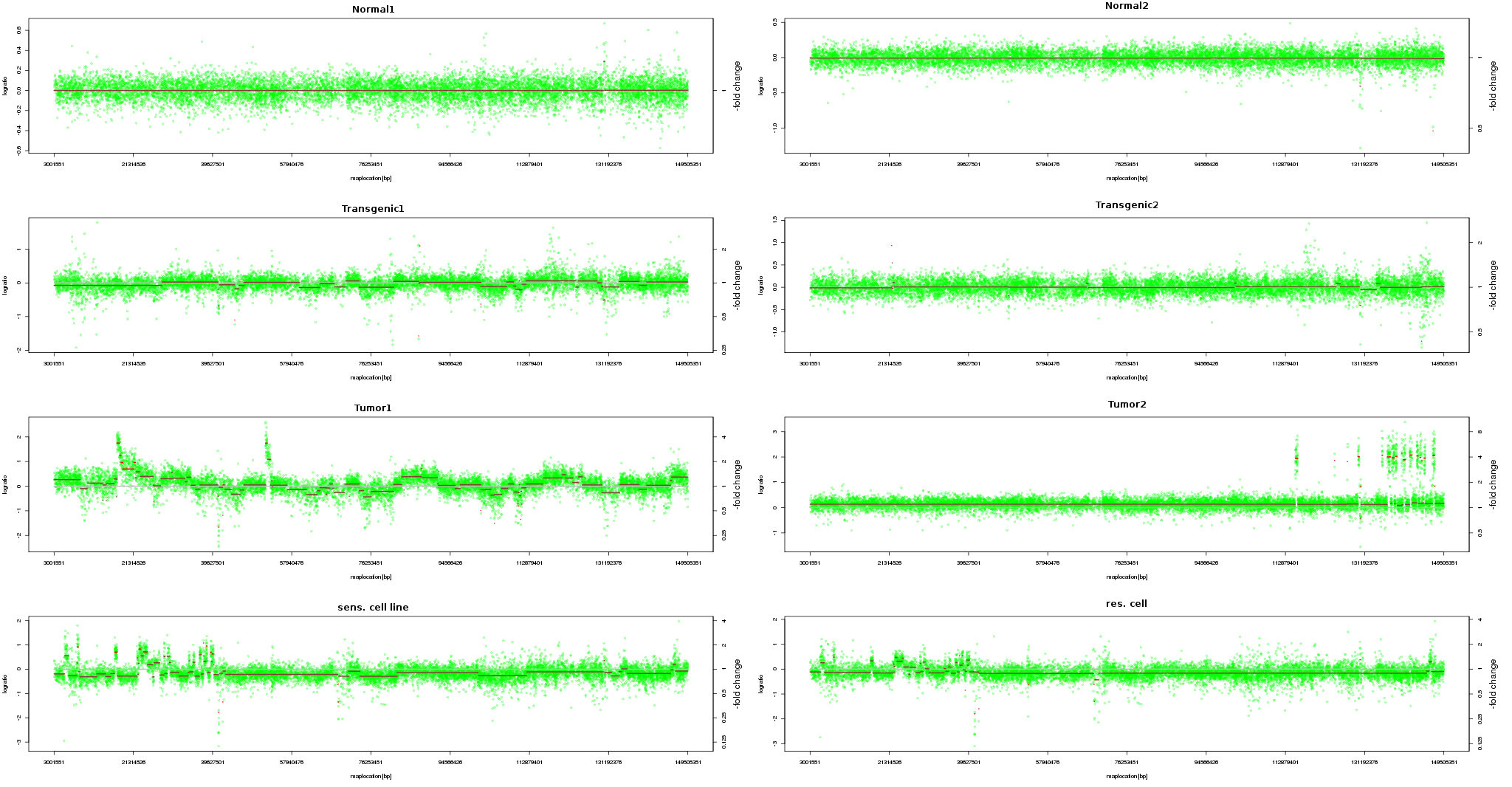

Supplement: Additional file 6 — Segmentation in different samples. Figure S3: Different segmentation results for chromosome 6 in all samples is depicted. Comparing Normal1 to Transgenic1 and to Tumor1, one can see an increase in both the fragmentation and the copy number. Comparable alterations can also be found in both SV40 cell line samples. By comparison, the Transgenic2 and Tumor2 samples show less fragmentations. Interestingly, even more segments can be identified in the Transgenic2 sample than in Tumor2. [file 1471-2407-12-380-S6.png]

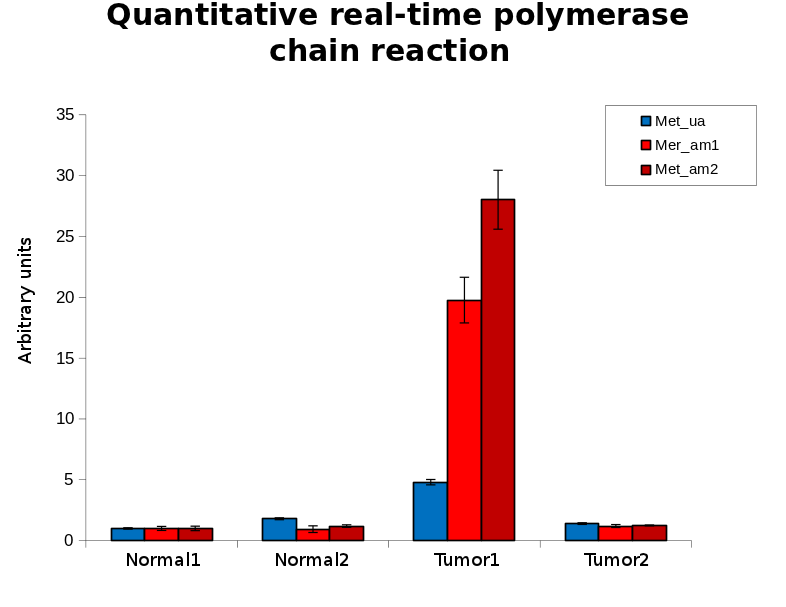

Supplement: Additional file 7 — Plot of qPCR results. Figure S2: Barplot illustrating the qPCR results for the three previously mentioned regions of chromosome 6. [file 1471-2407-12-380-S7.png]
