# Supplementary material for: SNP microarray analyses reveal copy number alterations and progressive genome reorganization during tumor development in SVT/t driven mice breast cancer
Source: BMC Cancer. 2012 Aug 31;12:380. doi: 10.1186/1471-2407-12-380 (PMC3534550; doi:10.1186/1471-2407-12-380)
Supplement: Additional file 2 — Kaplan-Meier curve. Figure S1: This figure illustrates the Kaplan-Meier curve. The x-axis depicts the duration from the first mating and the finding of tumor formation. All mice were euthanized as soon as a tumor was found. All 64 mice developed breast cancer within less than 200 days after their first day of pregnancy. In fact, about 60% of the animals showed a tumor formation within the first 100 days. [file 1471-2407-12-380-S2.pdf]

Table S1: Sample names used in this publication and GEO accession numbers for experimental data.

Trival name	Sample name	Description	Expression data	SNP data
Normal1	NORLAK III	NMRI mouse sample III first day of lactation	GSM143891	GSM876958
Normal2	NORLAK II	NMRI mouse sample II first day of lactation	GSM143330	GSM876959
Transgenic1	WAP-SVT-t mouse sample LAK I	first day of lactation	GSM143903	GSM876960
Transgenic2	WAP-SVT-t mouse sample LAK IV	the first day of lactation	*no data*	GSM876961
Tumor1	583 tumor 1	WAP-SVT/t breast cancer	GSM143908	GSM876962
Tumor2	597pos9 tumor	WAP-SVT/t breast cancer	GSM152129	GSM876963
res. cell line	SVTneg1 CAr	762TuD cell line sample cytosine arabinoside resistant	*no data*	GSM876965
sens. cell line	SVTneg1 CAs	762TuD cell line sample cytosine arabinoside sensitive	*no data*	GSM876964
RCT-*	-	Recurrent tumor samples by Liu et al. 2011	*no data*	GSE27691
