# Supplementary material for: SNP microarray analyses reveal copy number alterations and progressive genome reorganization during tumor development in SVT/t driven mice breast cancer
Source: BMC Cancer. 2012 Aug 31;12:380. doi: 10.1186/1471-2407-12-380 (PMC3534550; doi:10.1186/1471-2407-12-380)
Supplement: Additional file 3 — Number of segments computed for each sample. Table S2: This table lists the number of segments calculated for each chromosome in each of the 14 samples. [file 1471-2407-12-380-S3.pdf]

Table S3 (A): Percentage of log2 ratio values for single SNP probesets.

	x <= -0.6	-0.6 < x < -0.2	-0.2 <= x <= 0.2	0.2 < x < 0.6	x >= 0.6
Normal1	0.03	2.44	96.06	0.01	1.46
Normal2	0.04	2.62	95.91	0.00	1.42
Transgenic1	1.24	13.41	75.70	0.77	8.88
Transgenic2	0.57	9.52	80.18	0.51	9.22
Tumor1	4.55	16.66	58.22	1.37	19.21
Tumor2	1.04	19.14	60.65	1.26	17.90
res. cell line	3.34	22.73	55.01	1.85	17.07
send. cell line	3.73	22.52	53.43	2.12	18.20
RCT-D782	0.33	15.69	73.93	0.04	10.02
RCT-E565	0.20	12.35	79.03	0.09	8.33
RCT-E302	0.18	10.40	81.37	0.24	7.81
RCT-E473	0.08	8.89	81.19	0.07	9.77
RCT-D419	0.37	15.07	75.09	0.21	9.26
RCT-C658	0.11	8.09	83.64	0.0	8.06

Table S3 (B): Percentage of log2 ratio values for calculated segments.

	x <= -0.6	-0.6 < x < -0.2	-0.2 <= x <= 0.2	0.2 < x < 0.6	x >= 0.6
Normal1	7.14	8.57	75.71	7.14	1.43
Normal2	11.27	11.27	73.24	4.23	0.00
Transgenic1	11.53	9.96	72.87	1.70	3.93
Transgenic2	10.11	5.42	74.01	5.05	5.42
Tumor1	14.23	27.44	54.17	2.20	1.97
Tumor2	10.92	6.90	55.75	5.75	20.69
res. cell line	10.03	13.09	50.14	18.66	8.08
sens. cell line	4.14	15.41	60.34	15.55	4.57
RCT-D782	3.03	6.06	84.85	0.00	6.06
RCT-E565	0.00	7.69	87.18	0.00	5.13
RCT-E302	7.60	17.72	67.09	2.53	5.06
RCT-E473	5.62	20.23	66.29	6.74	1.12
RCT-D419	10.00	2.50	80.00	0.00	7.50
RCT-C658	0.00	24.64	65.22	4.35	5.80
