# Supplementary material for: SNP microarray analyses reveal copy number alterations and progressive genome reorganization during tumor development in SVT/t driven mice breast cancer
Source: BMC Cancer. 2012 Aug 31;12:380. doi: 10.1186/1471-2407-12-380 (PMC3534550; doi:10.1186/1471-2407-12-380)
Supplement: Additional file 4 — Copy number alteration and motif position in Tumor1 sample. Figure S4: Copy number alterations are depicted in the outer cirular plot. The five inner circular plots illustrate the motif positions of motif 1 (blue), motif 2 (orange), motif 3 (green), motif 4 (red), motif 5 (purple) and motif 6 (grey). Thicker lines illustrate a short distance of two motif positions. Common breakpoints of Tumor2 and Tumor1 samples are illustrated in the most inner circular plot. [file 1471-2407-12-380-S4.pdf]

Table S2: List of calculated segments in all samples

Chromosome	1	2	3	4	5	6	7	8	9	10	11	12	13	14	15	16	17	18	19	X	Sum
**Normal1**	1	7	5	3	1	3	11	4	1	8	1	3	3	1	1	1	1	1	3	5	64
**Normal2**	3	5	3	1	1	5	7	6	1	1	1	3	3	3	1	3	3	3	3	7	63
**Transgenic1**	54	67	44	35	33	39	38	43	32	41	27	37	32	18	38	39	39	14	7	77	754
**Transgenic2**	8	20	29	16	18	15	43	23	1	8	3	14	5	3	13	14	25	3	15	6	282
**Tumor1**	102	75	96	61	48	101	61	72	42	86	27	46	36	27	57	52	54	54	30	112	1239
**Tumor2**	7	7	5	10	5	37	13	9	5	7	5	9	8	1	7	7	11	9	9	3	174
**Common breakpoints Tu2 vs. Tu1**							4					3			1		2	2	1		
**res. cell line**	16	21	28	15	13	54	27	23	23	11	14	19	19	5	19	8	16	8	9	7	355
**sens. cell line**	53	44	46	38	41	69	53	38	39	37	27	30	17	23	29	20	45	24	12	7	692
**RCT-D782**	3	1	2	1	1	1	1	1	1	3	1	1	1	1	1	1	1	3	1	3	29
**RCT-E565**	1	1	1	3	3	8	1	1	1	1	1	1	1	1	1	1	1	3	1	3	35
**RCT-E302**	5	5	7	3	1	3	3	1	1	1	6	5	3	3	4	5	3	4	1	13	77
**RCT-E473**	7	10	5	4	5	3	3	5	1	3	2	3	7	1	7	3	3	3	1	9	85
**RCT-D419**	1	1	1	1	3	3	1	3	1	1	1	1	3	1	5	1	1	3	1	5	38
**RCT-C658**	1	1	3	1	5	1	1	11	1	3	1	1	7	2	18	1	1	3	1	1	64
