# Supplementary material for: SNP microarray analyses reveal copy number alterations and progressive genome reorganization during tumor development in SVT/t driven mice breast cancer
Source: BMC Cancer. 2012 Aug 31;12:380. doi: 10.1186/1471-2407-12-380 (PMC3534550; doi:10.1186/1471-2407-12-380)
Supplement: Additional file 8 — Genotyping Protocol. Protocol of genotyping analyses. [file 1471-2407-12-380-S8.pdf]

Protocol of Affymetrix Mapping Assay

Genotyping experiments were performed by ATLAS Biolabs GmbH Berlin. This protocol lists procedures for genotyping analyses using the Affymetrix Mouse Diversity Genotyping array.

Labeling of genomic DNA and hybridization

Genomic DNA was labelled according to the Affymetrix Genome-Wide Human SNP Nsp/Sty 6.0 User Guide (P/N 702504, Rev 3). 500 nanograms of genomic DNA were used as starting material.

Hybridization of labelled genomic DNA and washing

Hybridization conditions: 50C, 60 rpm, 16-18 h; in an hybridization oven (Hybridization Oven 640, Affymetrix). Washing and staining was performed with a fluidics station (GeneChip Fluidics Station 450, Affymetrix), which is controlled by Affymetrix software GeneChip Command Console v2.0.

Scanning of arrays and primary data analysis

Scanning was performed with the GeneChip Scanner 3000 7G (Affymetrix), which is controlled by Affymetrix software GeneChip Command Console v2.0. Primary data analysis was performed using Affymetrix Genotyping Console v4.1.
